# Gradients of metabolite accumulation and redifferentiation of nutritive cells associated with vascular tissues in galls induced by sucking insects

**DOI:** 10.1093/aobpla/plv086

**Published:** 2015-07-24

**Authors:** Renê Gonçalves da Silva Carneiro, Rosy Mary dos Santos Isaias

**Affiliations:** Departamento de Botânica, Instituto de Ciências Biológicas, Universidade Federal de Minas Gerais, Av. Antônio Carlos, 6627, Belo Horizonte, Minas Gerais, Brazil

**Keywords:** Carbohydrate-related enzyme activity, cytology, development, gall, metabolic gradients, primary and secondary metabolites, *Psidium cattleianum*

## Abstract

Plant cells respond to abiotic and biotic stimuli, which generate adaptive phenotypes in plant organs. In the case of plant galls, cell phenotypes are adaptive for the gall inducer and assume characteristics mainly linked to its protection and nutrition. Herein, the cytological development and histochemical profile of *Nothotrioza cattleiani* galls, a sucking insect, on the leaves of *Psidium cattleianum* are compared with those of other galls, especially *N. myrtoidis* galls, searching for conserved and divergent alterations in cell fates and cycles. Leaf cell fates are completely changed within galls, except for epidermal cells, but the comparison between *Nothotrioza* spp. galls shows conserved fates. Nevertheless, cytological development of *N. cattleiani* galls is different from the standby-redifferentiation of *N. myrtoidis* galls. Starch and lignins, and reducing sugars form centrifugal and centripetal gradients of accumulation, respectively. Proteins, total phenolics, terpenoids, proanthocyanidins and reactive oxygen species are detected in bidirectional gradients, i.e. weak or undetectable reaction in the median cortical cells that is gradually more intense in the cell layers towards the inner and outer surfaces of the gall. True nutritive cells associated with vascular tissues, together with the bidirectional gradients of metabolite accumulation, are herein reported for the first time in insect galls. The globoid galls of *N. cattleiani*, though macro-morphologically similar to the galls of *N. myrtoidis*, are distinct and unique among insect galls, as far as the cellular, subcellular and histochemical traits are concerned. Thus, the traits of the galls on *P. cattleianum* studied herein represent the extended phenotypes of their inducers.

## Abstract (precis)

Galls are structures entirely made of plant tissues manipulated by gall-inducing parasites. We analyzed the alterations of cell phenotypes caused by a sucking-insect, *Nothotrioza cattleiani*, on the leaves of *Psidium cattleianum* that led to the formation of a globoid gall morphotype. We found changed cell fates in the leaves compared to the galls, and different degrees of cell alterations in gall layers, which formed gradients. Surprisingly, the vascular parenchyma cells on *N. cattleiani* galls are nutritive. Herein, we show that even though the globoid shape is one of the most common in nature, the galls of *N. cattleiani* are unique entities.

## Introduction

The capability of insects to induce galls depends on variable properties of plant cells, which are ultimately defined by the biotic and abiotic stimuli from the surrounding environment, and lead to different cell phenotypes (Gianoli and Valladares 2012). Plant cells under the influence of gall-inducing insects redifferentiate (*sensu* Lev-Yadun 2003) and often assume rapid cell cycles and new cell fates for the neo-ontogenesis of plant galls (*sensu* Carneiro *et al*. 2014*a*). Even though specific galling insect taxa are classically known to induce conserved, morphologically different galls (Mani 1964; Rohfritsch 1992), recent studies have documented extremely diverse aspects of cell biology in neotropical galls (Moura *et al*. 2009; Oliveira and Isaias 2009, 2010*a*; Formiga *et al*. 2011; Isaias *et al*. 2011; Castro *et al*. 2012*a*; Bedetti *et al*. 2013; Dias *et al*. 2013; Ferreira and Isaias 2013; Guimarães *et al*. 2013, 2014).

The metabolism of certain neotropical gall taxa has been assessed using cytological and histochemical methods (Oliveira and Isaias 2010*b*; Oliveira *et al*. 2010, 2011*a*, *b*; Vecchi *et al*. 2013) in the search for gradients already described for nutritive tissues in Cynipidae and Cecidomyiidae galls (Bronner 1992). In the Neotropical galls with true nutritive tissues, some of the metabolic gradients proposed by Bronner (1992) were corroborated (Oliveira *et al*. 2010, 2011*b*). Unexpectedly, cytological and histochemical gradients, i.e. increasing degree of cell alterations and of metabolite accumulation in the cortical cells of the gall towards the larval chamber, are also reported for the galls induced by the sucking insect, *Pseudophacopteron* sp. (Psyllidae), on *Aspidosperma australe* (Oliveira and Isaias 2010*b*), which have nutritive-like parenchyma cells. Despite being regarded as structurally simple and devoid of nutritive cells (Meyer 1987), galls of sucking insects, namely *Euphalerus ostreoides* (Isaias *et al*. 2011) and *Nothotrioza myrtoidis* (Carneiro and Isaias 2014; Carneiro *et al*. 2014*b*), also have cytological and histochemical characteristics of metabolically active cells. These cells are evidenced in the vascular tissues and parenchyma near the nymphal chamber. *Nothotrioza myrtoidis* galls have gradients of primary and secondary metabolites, discrete accumulation of reactive oxygen species (ROS) and are photosynthesis deficient (Carneiro *et al*. 2014*b*). Moreover, the completion of cell cycles through standby-redifferentiation is ensured by the chemical (Carneiro *et al*. 2014*b*) and cytological ROS-scavenging apparatus (Carneiro and Isaias 2014).

Herein, we assess the cytology and histochemistry of the galls induced by *Nothotrioza cattleianum* Burckhardt (Triozidae) (Carneiro *et al*. 2013) on *Psidium cattleiani* Sabine (Myrtaceae). The structural–functional aspects related to cell biology in *P. cattleianum* galls are compared with those of other galls, especially the co-generic system of *N. myrtoidis*–*P. myrtoides*. The galls of *Nothotrioza* spp. on *Psidium* spp. are both globoid (*sensu* Isaias *et al*. 2013) and have similar phenology (Butignol and Pedrosa-Macedo 2003; Carneiro *et al*. 2013). We expect that the developmental cytology and histochemical profile of *P. cattleianum* galls should reveal conserved traits with regards to the sucking feeding habit of *N. cattleiani* and also distinctive ones of a unique extended phenotype (*sensu* Bailey *et al*. 2009). The following questions are addressed: (i) Do the cells of *N. cattleiani* galls have conserved cell fates in relation to the non-galled leaves, and in comparison to *N. myrtoidis* galls? (ii) Do the cytological and histochemical profiles of *Nothotrioza* spp. galls indicate unique neo-established tissue functionalities?

## Methods

### Sampling

The population of *P. cattleianum* Sabine (Myrtaceae) with galls induced by *N. cattleiani* Burckhardt is located at the Parque Estadual Pico do Marumbi, municipality of Piraquara, Paraná state, Brazil. Non-galled leaves (young and mature, *n* = 5 per developmental stage) and galls at the phases of induction, growth and development, maturation, and senescence (*n* = 5 per developmental stage) were collected during the years 2012 and 2013. The samples were fixed in 2.5 % glutaraldehyde (Grade I) and 4.5 % formaldehyde in phosphate buffer (0.1 M; pH 7.2) (Karnovsky 1965).

### Light microscopy

Fixed samples were dehydrated in ethanol series (Johansen 1940), embedded in glycolmethacrylate (Leica^®^), sectioned (6–10 µm) with a rotary microtome Hyrax (Zeiss^®^) and stained with 0.05 % toluidine O blue (pH 4.6) (O'Brien *et al*. 1964). Histological slides were observed and photographed using a light microscope (Leica^®^ DM500) coupled with a digital camera (Leica^®^ ICC50 HD).

### Histochemical analysis

Histochemical tests for primary and secondary plant metabolites, ROS and the activity of enzymes related to carbohydrate metabolism were performed using fresh samples of mature leaves and galls. Control tests were performed according to the references and also by comparison with blank sections. Samples were submitted to the tests/reagents, following the methods described subsequently.

#### Fehling's reagent (reducing sugars)

Equal parts of ‘A’ (copper(II) sulfate 6.93 % w : v) and ‘B’ (sodium potassium tartrate 34.6 and 12 % sodium hydroxide w : w : v) solutions and heat to pre-boiling temperature (Sass 1951).

#### Lugol's reagent (starch)

1 % potassium iodine–iodide solution for 5 min (Johansen 1940).

#### Sudan red B (total lipids)

Saturated solution of Sudan red B in 70 % ethanol for 5 min (Brundrett *et al*. 1991).

#### Coomassie blue (total proteins)

0.25 % Coomassie blue solution for 5 min (Dunn 1993).

#### Ferric chloride (phenolics)

1 % ferric chloride solution for 5 min (Johansen 1940).

#### *p*-Dimethylaminocinnamaldehyde (proanthocyanidins)

Fixation in 0.5 % caffeine sodium benzoate in 90 % butanol for 1–2 h. Reaction in 1 % *p*-dimethylaminocinnamaldehyde (DMACA) (Feucht *et al*. 1986).

#### Nadi reagent (terpenoids)

1 % α-Naphtol, 1 % dimethyl-*p*-phenylenediamine in 0.01 M phosphate buffer (pH 7.2) for up to 30 min (David and Carde 1964).

#### Wiesner reagent (lignins)

2 % Phloroglucinol in acidified solution for 5 min (Johansen 1940).

#### DAB reagent (ROS)

0.5 % 3,3′-diaminobenzidine (DAB) for 30 min in the dark (Rossetti and Bonatti 2001).

#### Acid phosphatase activity

Incubation in 0.012 % lead nitrate and 0.1 M potassium sodium glycerophosphate in 0.5 M acetate buffer (pH 4.5) for 24 h, at room temperature. Reaction in 1 % ammonium sulfate for 5 min, after washing in distilled water (Gomori 1956).

#### Glucose-6-phosphatase activity

Incubation in 20 mg of potassium glucose-6-phosphate in 125 mL of 0.2 M Tris–maleate buffer (pH 6.7), 3 mL of 2 % lead nitrate in 7 mL of distilled water for 15 min to 2 h, at 37 °C. Reaction in 1 % ammonium sulfate for 5 min, after washing in distilled water (Jensen 1962).

#### Phosphorylase activity

Incubation in 1 % glucose-1-phosphate in 0.1 M acetate buffer (pH 6.0) for 2 h at room temperature. Reaction in Lugol's reagent for 5 min (Jensen 1962).

#### Sucrose synthase activity

Fixation in 2 % paraformaldehyde with 2 % polyvinylpyrrolidone and 0.005 M dithiothreitol for 1 h at 4 °C. Incubation in 5 mL of 150 mM NADH, 5 mL (1 U) of phosphoglucomutase, 5 mL of 3 mM glucose-1,6-biphosphate, 5 mL (1 U) of glucose-6-phosphate dehydrogenase, 5 mL (1 U) of uridine diphosphate glucose–pyrophosphorylase, 280 mL of 0.07 % aqueous nitroblue tetrazolium (NBT), 350 mL of buffer and 50 mL of substrate for 30 min. Buffer comprised 100 mM HEPES, 10 mM MgCl_2_, 2 mM ethylenediamine tetraacetic acid, 0.2 % bovine serum albumin, 2 mM ethylene glycol tetraacetic acid at pH 7.4. Substrate is composed of 0.75 M sucrose, 15 mM uridine diphosphate and 15 mM pyrophosphate (Wittich and Vreugdenhil 1998).

#### Invertases activity

Incubation in 0.38 mM sodium phosphate (pH 7.5), 0.024 % NBT, 0.014 % phenazine methosulfate, 30 U of glucose oxidase and 30 mM of sucrose at room temperature for 3 h (Doehlert and Felker 1987; Zrenner *et al*. 1995).

Treated sections were mounted on glass slides with Kaiser's glycerol gelatin (Kraus and Arduin 1997), observed and photographed with a light microscope (Leica^®^ DM500) coupled with a digital camera (Leica^®^ ICC50 HD).

### Transmission electron microscopy

The fixed samples were post-fixed in 1 % osmium tetroxide in phosphate buffer (0.1 M; pH 7.2), dehydrated in ethanol series (O'Brien and McCully 1981) and embedded in Spurr's^®^ resin. Ultrathin sections were obtained with a diamond knife in the Reichert–Jung Ultracut ultramicrotome (Leica, Wetzlar, Germany), attached to copper grids, contrasted with uranyl acetate and lead citrate (Reynolds 1963). The sections were analysed using a transmission electron microscope Tecnai™ G2-12—SpiritBiotwin (FEI, Hillsboro, USA) at 120 kV, at the Centro de Microscopia of the Universidade Federal de Minas Gerais (CM-UFMG).

## Results

### Morphology and development of leaves and galls

**Figure 1.**
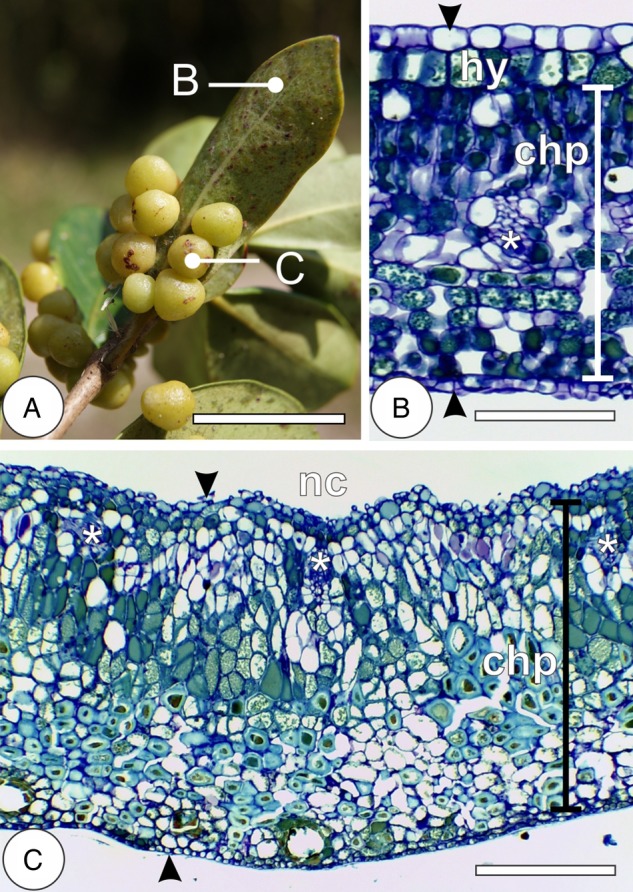
Morphology and anatomy of *Psidium cattleianum* leaves and *Nothotrioza myrtoidis* galls. (A) The detail of a simple leaf with globoid galls protruded to the abaxial surface. (B) Cross-section of mature leaf with uniseriate epidermis on both surfaces (arrowheads), hypodermis (hy) and vascular bundles (asterisks) interspaced to the dorsiventral chlorophyllous parenchyma (chp). (C) Cross-section of mature gall with uniseriate epidermis on both surfaces (arrowheads) and vascular bundles (asterisks) near the nymphal chamber (nc) interspaced to the homogenous chlorophyllous parenchyma (chp). Bars: (A) 3 cm; (B) 100 µm; (C) 200 µm.

The galls of *N. cattleiani* are globoid and protruded to the abaxial surface of the leaves of *P. cattleianum* (Fig. 1A). The young leaves of *P. cattleianum* have uniseriate epidermis, homogenous chlorophyllous parenchyma with interspaced vascular bundles undergoing differentiation. As they mature, epidermis remains uniseriate; hypodermis differentiates under the adaxial surface of the epidermis, and dorsiventral chlorophyllous parenchyma, interspaced with collateral vascular bundles, is observed (Fig. 1B). The galls of *N. cattleiani* are induced on the young leaves, whose cells redifferentiate to form a depression on the leaf lamina, and ultimately generate a globoid gall with an ample chamber and relatively thin wall. In mature galls, such walls have uniseriate inner and outer epidermis, hyperplasic and hypertrophied homogenous parenchyma and collateral vascular bundles near the nymphal chamber (Fig. 1C). The galls have a 1-year life cycle, with four developmental stages: induction, growth and development, maturation and senescence.

### Cytological development of cell lineages

#### Epidermis

**Figure 2.**
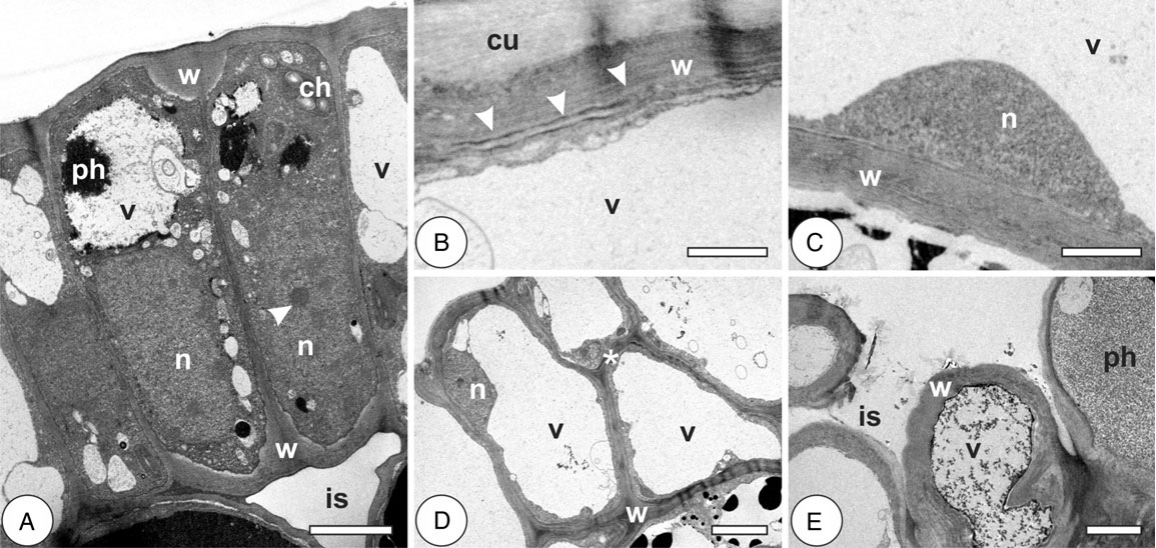
Cytology of epidermal cells in the leaves of *Psidium cattleianum* and galls of *Nothotrioza cattleiani*. (A–C) Leaves. (D and E) Galls. (A) Cells with thin anticlinal primary walls (w), large nuclei (n) with conspicuous nucleoli (arrowhead), few chloroplasts (ch) and small vacuoles (v), with phenolic inclusions (ph). (B) The detail of a cell with polylamellate (arrowheads) cell wall (w), thick cuticle (cu) and hyaline vacuole (v). (C) The detail of a cell with homogenous secondary wall (w), small nucleus (n) and hyaline vacuole (v). (D) Induction phase. Cells with homogenous thickened walls (w), peripheral nuclei (n) and hyaline vacuoles (v). Discrete sites of periclinal divisions are observed (asterisk). (E) Maturation phase. Intermittent cell layer, with intercellular spaces (is), heterogeneous thickened and polylamellate walls and inconspicuous cuticle. Vacuoles (v) may contain phenolic inclusions (ph). Bars: (A–C) 2 µm; (D and E) 5 µm.

In the young leaves of *P. cattleianum*, epidermal cells have thin anticlinal walls, fragmented hyaline vacuoles, few chloroplasts and mitochondria, and large central nuclei with conspicuous nucleoli (Fig. 2A; Table 1). In the mature leaves, the walls are thicker, and the vacuoles have confluenced into a big central vacuole with phenolics; chloroplasts and mitochondria are rare, and the nuclei are small with peripheral heterochromatin (Fig. 2B and C). Galls are induced on young leaves, and exhibit epidermal cells with homogenously thin walls, with large hyaline vacuoles. Nuclei are peripheral and the electron-dense cytoplasm is reduced to a fine region near the cell walls (Fig. 2D). Epidermal cells in the galls at the stage of growth and development have heterogeneous thick cuticle, with protoplast similar to that of previous stage. At the phase of maturation, the inner epidermis is intermittent; cells exhibit heterogeneously thickened and polylamellate walls and thin cuticle (Fig. 2E; Table 1). The protoplast is poorly altered in comparison to the cells of the previous stage. At senescence, the cells have vacuoles with phenolic inclusions and walls with disaggregated lamellae and end up undergoing autolysis.

**Table 1.** Structural and ultrastructural traits of the leaves of *Psidium cattleianum* and galls induced by *Nothotrioza cattleiani*.

Tissues	Leaves	Galls
Epidermis	Uniseriate; continuous cell layer; low metabolic apparatus	Uniseriate; discontinuous cell layer (inner epidermis); low metabolic apparatus
Chlorophyllous parenchyma	Dorsiventral; cells with well-developed metabolic apparatus; many large plastoglobules	Homogenous; cells with underdeveloped metabolic apparatus; few small plastoglobules; lamellar and multivesicular bodies
Vascular and perivascular parenchyma	Collateral vascular bundles; cells with developed respiratory and protein synthesis apparatus	Collateral vascular bundles; cells with highly developed respiratory and protein synthesis apparatus; lomasomes, lamellar and multivesicular bodies

#### Chlorophyllous parenchyma

**Figure 3.**
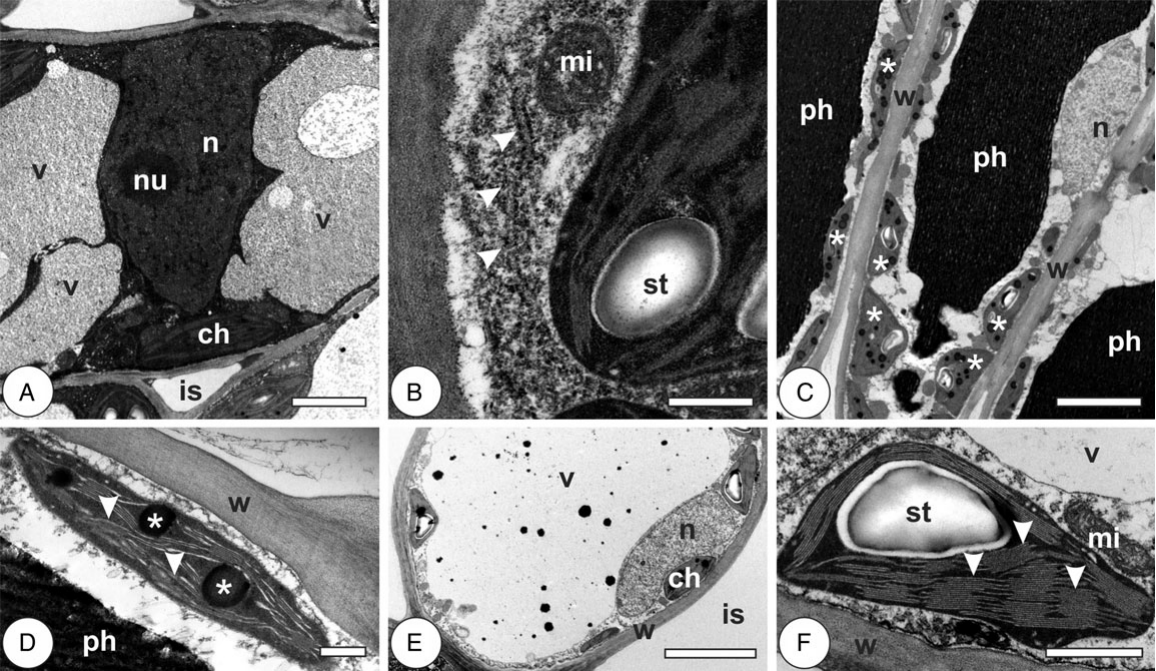
Cytology of chlorophyllous parenchyma cells in leaves of *Psidium cattleianum*. (A and B) Young leaves. (C–F) Mature leaves. (A) Cell with primary thin walls, large central nucleus (n) with conspicuous nucleolus (nu), fragmented vacuole (v) and chloroplasts (ch) with well-developed grana. Intercellular spaces (is) are reduced. (B) The detail of a cell with dense cytoplasm, RER (arrowheads) and mitochondria (mi) associated with chloroplasts with well-developed grana and starch grain (st). (C) Palisade parenchyma cells with homogenous walls (w), phenolic-rich vacuoles (ph), many chloroplasts with well-organized thylakoid lamellae and starch grains (asterisks) and peripheral nuclei (n) with inconspicuous nucleoli. (D) The detail of a chloroplast with low-stack grana (arrowheads) and large plastoglobules (asterisks). (E) Spongy parenchyma cell with homogenous primary wall (w), large vacuole (v), peripheral nucleus (n), chloroplasts (ch) and large intercellular space (is). (F) The detail of a chloroplast with well-developed thylakoid system, high-stack grana (arrowheads), starch grain (st) and associated mitochondria (mi). Bars: (A) 2 µm; (B and D) 500 nm; (C and E) 5 µm; (F) 1 µm.

In the young leaves, mesophyll cells have thin, homogenous primary cell walls and reduced intercellular spaces. Their vacuoles are large, sometimes fragmented around the nucleus, with electron-dense granular inclusions. Nuclei are large, with electron-dense euchromatin and conspicuous nucleoli (Fig. 3A). Chloroplasts are elliptical, with organized grana, small starch grains and scarce electron-dense plastoglobules. Small round mitochondria are in association with chloroplasts, and rough endoplasmic reticulum (RER) is sometimes observed on the periphery of the cytoplasm (Fig. 3B; Table 1). In mature leaves, the palisade parenchyma cells have large phenolic-rich vacuoles, many chloroplasts with well-organized thylakoid lamellae and starch grains; nuclei are peripheral, with inconspicuous nucleoli (Fig. 3C). Chloroplasts with large and numerous plastoglobules are frequent (Fig. 3D). Spongy parenchyma cells have large hyaline vacuoles, peripheral nuclei with inconspicuous nucleoli and chloroplasts with well-organized thylakoid lamellae, starch grains and rare plastoglobules (Fig. 3E and F).

**Figure 4.**
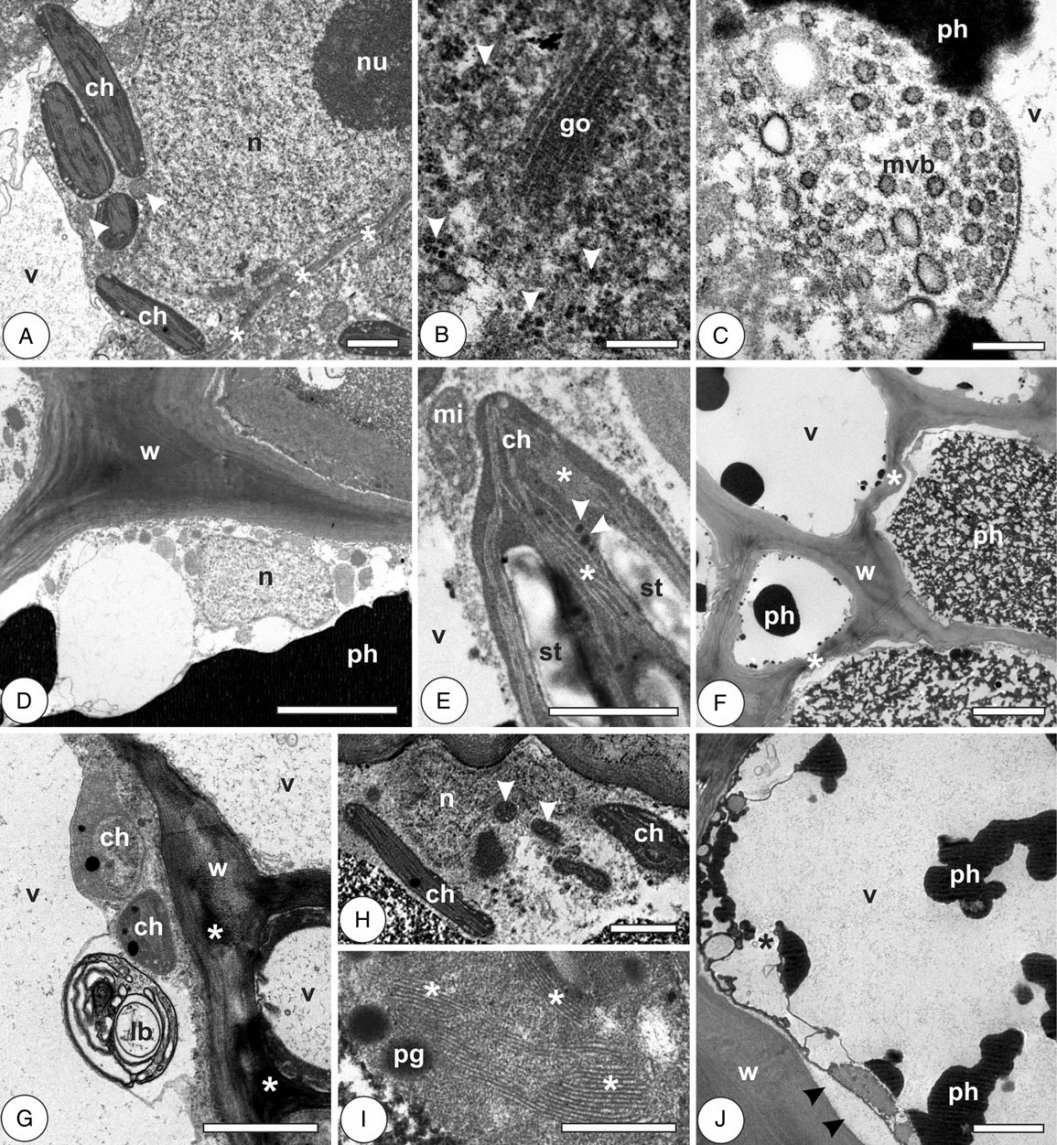
Cytology of chlorophyllous parenchyma cells in the galls of *Nothotrioza cattleiani*. (A–C) Induction phase. (D and E) Growth and development phase. (F–H) Maturation phase. (I and J) Senescent phase. (A) The detail of a cell with large nuclei (n) with conspicuous nucleoli (nu), small mitochondria (arrowheads) associated with chloroplasts (ch) with well-organized lamellae. (B) Abundant polysomes (arrowheads) and Golgi apparatus (go). (C) Multivesicular body (mvb) at cytoplasm periphery. (D) Cells with secondary polylamellate walls (w), phenolic-rich vacuoles (ph) and small nuclei (n) with inconspicuous nucleoli. (E) Mitochondria (mi) associated to chloroplast (ch) with well-organized thylakoid lamellae (asterisks), starch grains (st) and small plastoglobules (arrowheads). (F) Cells with irregularly thickened walls (w, asterisks), phenolic (ph) or hyaline vacuoles (v). (G) The detail of lamellar body (lb), undeveloped chloroplasts (ch) and cell walls (w) with sites of different electron density (asterisks). (H) Degraded nucleus (n), small mitochondria (arrowheads) and chloroplasts. (I) Degraded chloroplast with vestigial grana (asterisks) and plastoglobules (pg). (J) Cell with large periplasmic spaces (arrowheads), disrupted vacuole (v, asterisk) with phenolic inclusions (ph). Bars: (A, E and H) 1 µm; (B and C) 200 nm; (D and F) 5 µm; (G and J) 2 µm; (I) 500 nm.

Galls at induction stage have hypertrophic and hyperplasic cells, with thin primary walls, sometimes fragmented vacuoles, with phenolic inclusions. They have large nuclei with conspicuous nucleoli, small mitochondria associated with chloroplasts with well-organized thylakoid lamellae and few small plastoglobules (Fig. 4A). Polysomes, Golgi apparatus and multivesicular bodies are sometimes visualized in the periphery of the cells (Fig. 4B and C). At the phase of growth and development, the cells have secondary polylamellated walls, phenolic-rich vacuoles and electron-lucent cytoplasm poor in organelles. Nuclei are small, with little heterochromatin at the periphery; mitochondria are small, associated with chloroplasts with well-organized thylakoid lamellae, starch grains and few small plastoglobules (Fig. 4D and E). Cells of mature galls are large, with big phenolic or hyaline vacuoles, reduced cytoplasm, sometimes with periplasmic spaces. Cell walls are secondary, either homogeneously thickened and polylamellate or irregularly thickened, sometimes polylamellate, with sites of different electron density (Fig. 4F and G). Organelles are scarce; lamellar bodies, degraded nuclei and small chloroplasts with underdeveloped thylakoid lamellae and plastoglobules are observed at the periphery of the cells (Fig. 4G and H). At gall senescence, the outer cortical cells exhibit thickened, polylamellate secondary walls and remain alive, with phenolic-rich vacuoles. Inner cortical cells have signs of degradation, with large periplasmic spaces, disrupted tonoplast and chloroplasts with vestigial thylakoid grana and plastoglobules (Fig. 4I and J; Table 1).

#### Vascular and perivascular parenchyma

**Figure 5.**
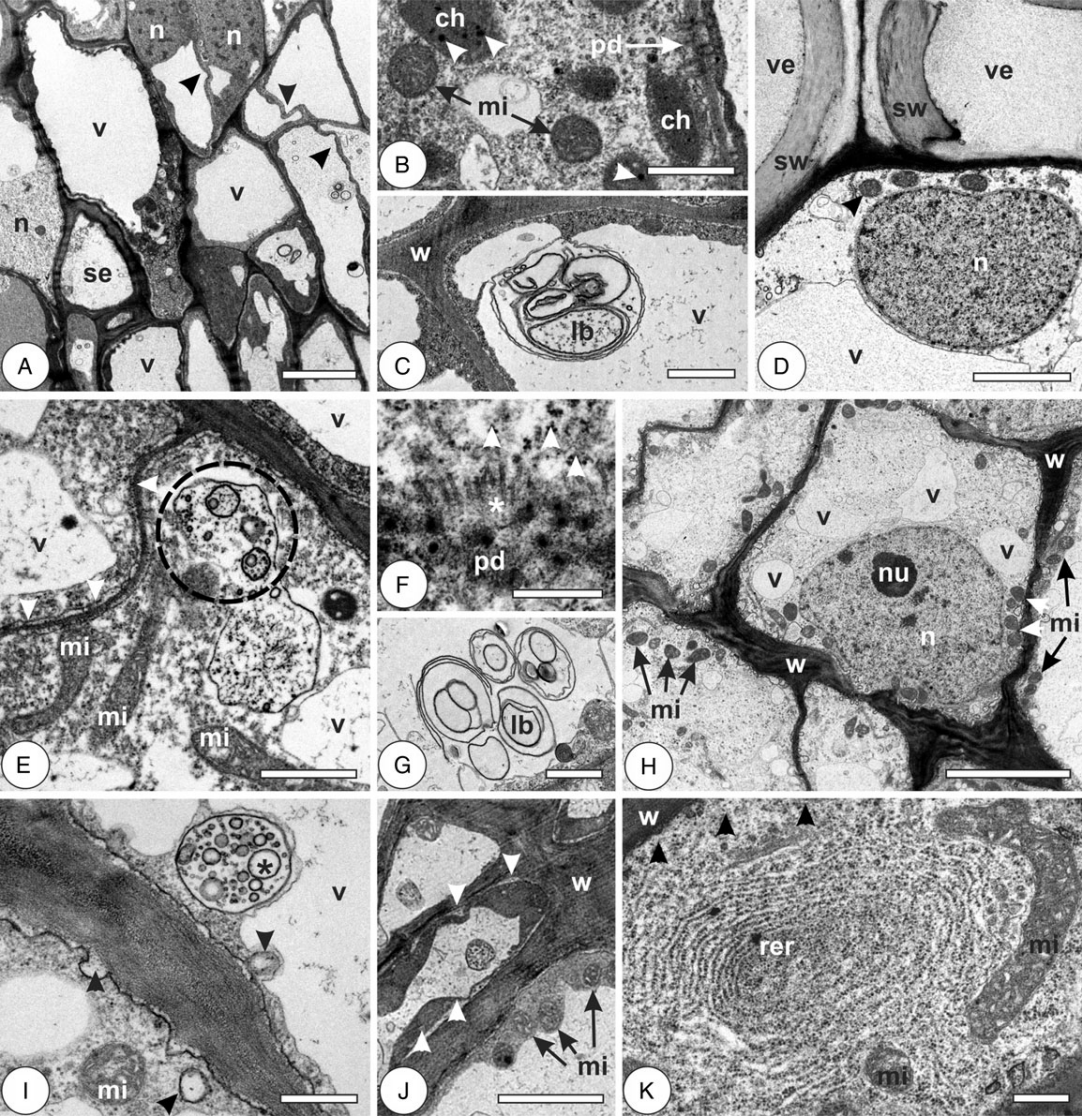
Cytology of vascular and perivascular parenchyma cells in the leaves of *Psidium cattleianum* and galls of *Nothotrioza cattleiani*. (A–C) Young leaves. (D) Mature leaves. (E–K) Galls. (A) Cells with thin and sinuous walls (arrowheads), large nuclei (n) and hyaline vacuoles (v). Sieve elements (se) may be observed. (B) Cell with dense cytoplasm, many plasmodesmata (pd), round mitochondria (mi) and small underdeveloped chloroplasts (ch) with few small plastoglobules (arrowheads). (C) Lamellar body (lb) near the cell wall (w). (D) Cell with large nucleus (n) with dispersed heterochromatin, mitochondria (arrowhead) and hyaline vacuole (v). Vessel elements (ve) with secondary cell walls (sw) can be observed. (E–G) Induction phase. (E) Cells with thin sinuous walls (arrowheads), hyaline vacuoles (v), large mitochondria (mi) and multivesicular bodies (dashed circle). (F) The detail of plasmodesmata (pd) with aligned microtubules (asterisks) and abundant polysomes (arrowheads). (G) The detail of lamellar bodies (lb). (H and J) Growth and development phase. (H) Cells with irregularly thickened walls (w), fragmented hyaline vacuoles (v), large nuclei (n) with conspicuous nucleoli (nu) and abundant mitochondria (mi). (I) The detail of cells with multivesicular bodies (asterisk) and lomasomes (arrowheads). (J) Cells during maturation phase, with thick walls (w), abundant mitochondria (mi) and periplasmic phases (arrowheads). (K) Cell during senescent phase, with abundant rough endoplasmic reticulum (rer), polysomes (arrowheads) and large mitochondria (mi). Bars: (A and H) 5 µm; (B, C, E and G) 1 µm; (D and J) 2 µm; (F, I and K) 500 nm.

In the young leaves, the cells have thin primary walls, sometimes sinuous and with many plasmodesmata; nuclei are large, with inconspicuous nucleoli and dispersed heterochromatin (Fig. 5A). They have large, sometimes fragmented hyaline vacuoles, dense cytoplasm with rounded mitochondria, small underdeveloped chloroplasts, with few plastoglobules, little RER and lamellar bodies continuous with the plasma membrane (Fig. 5B and C; Table 1). In mature leaves, cells have thin primary walls; cytoplasm is hyaline, with many mitochondria, scarce RER and large nuclei with dispersed heterochromatin (Fig. 5D). Phenolic-rich vacuoles and periplasmic spaces are frequent. In the galls at induction phase, cells are thin walled, with electron-dense cytoplasm, large nuclei, abundant polysomes, sometimes evident Golgi apparatus, large mitochondria, abundant plasmodesmata with evident microtubules (Fig. 5E and F). Lamellar and multivesicular bodies are frequent (Fig. 5G). At the phase of growth and development, cells have irregularly thickened walls with sites of different electron density and fragmented hyaline vacuoles. Cytoplasm is electron-lucent and organelle-rich, with abundant mitochondria and polysomes, Golgi apparatus, RER, large nuclei sometimes with conspicuous nucleoli and dispersed heterochromatin (Fig. 5H). Lomasomes and multivesicular bodies are frequent (Fig. 5I). Cells of mature galls have thickened and somewhat sinuous secondary walls, large vacuoles either hyaline or phenolic-rich, and abundant mitochondria. Cytoplasm is electron dense, and periplasmic spaces may be observed (Fig. 5J). At gall senescence, cells are metabolically active, with organelle-rich cytoplasm. They have abundant mitochondria, polysomes and RER; nuclei are large, with peripheral heterochromatin. Lomasomes and lamellar bodies are rarely seen (Fig. 5K; Table 1).

### Histochemical profile of leaves and galls

#### Primary metabolites

**Figure 6.**
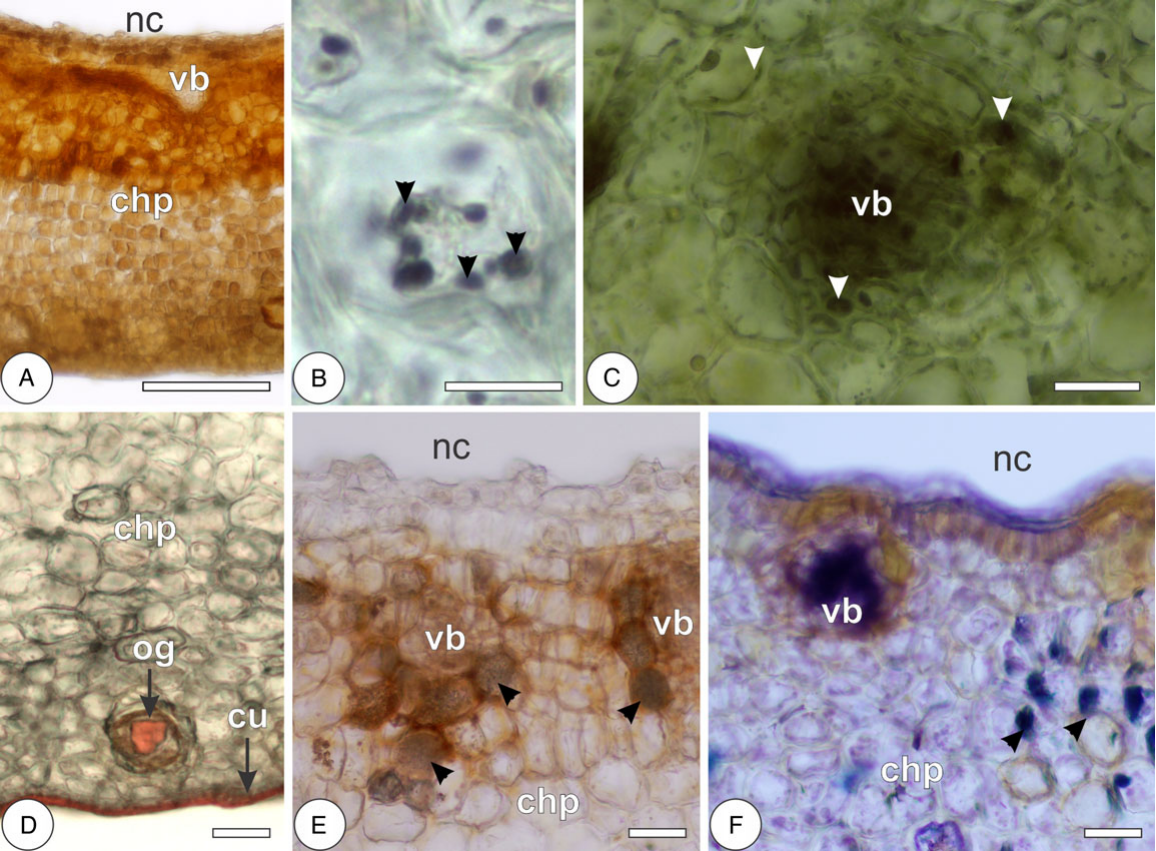
Histochemical detection of primary metabolites and carbohydrate-related enzyme activity in the galls of *Nothotrioza cattleiani*. (A) Reaction of Fehling's reagent to reducing sugars in the chlorophyllous parenchyma (chp), with increased accumulation near the vascular bundles (vb) and tissues near the nymphal chamber (nc). (B) Reaction of Lugol's reagent to starch (arrowheads) in outer cortical cells. (C) Reaction of Coomassie blue to proteins (arrowheads) in a vascular bundle (vb) and perivascular parenchyma. (D) Reaction of Sudan red B to lipids in the oil glands (og) and cuticle (cu) of the outer epidermis. (E and F) Enzyme activity of glucose-6-phosphatase and invertases, respectively, near vascular bundles (vb) and perivascular parenchyma (arrowheads), surrounding the nymphal chamber (nc). Bars: (A) 200 µm; (B) 10 µm; (C) 30 µm; (D–F) 60 µm.

In the leaves, reducing sugars and primary starch grains are, respectively, localized by Fehling's and Lugol's reagents throughout the chlorophyllous parenchyma and more rarely in the hypodermis. Reducing sugars are also localized in the phloem of vascular bundles. Lipid droplets and protein precipitates, respectively, stained by Sudan red B and Coomassie blue are observed more intensely in the chlorophyllous parenchyma and vascular bundles, and more scarcely in the epidermal cells and oil glands. The cuticle on both epidermal surfaces and the secretion of oil glands are also stained by Sudan red B. In the galls, reducing sugars are strongly stained by Fehling's reagent, forming a centripetal gradient of sugar accumulation from the outermost cell layers towards the inner cortex (Figs 6A and 8; Table 2). Primary starch grains are rarely stained, but they may form a discrete centrifugal gradient evidenced by the reaction to Lugol's reagent (Figs 6B and 8). Protein precipitates are stained by Coomassie blue in the vascular bundles, perivascular parenchyma and outermost cortical cell layers, forming a bidirectional gradient from the median cortex towards the inner and outer cortices (Figs 6C and 8). The cuticle of the outer epidermal cells and the secretion of oil glands were stained by Sudan red B (Fig. 6D; Table 2).

**Table 2.** Histochemical traits of the leaves of *Psidium cattleianum* and galls induced by *Nothotrioza cattleiani*.

Detected substances	Reaction sites	
	Leaves	Galls
Reducing sugars	Chlorophyllous parenchyma and vascular tissues	Centripetal gradient (weakly in the outermost cell layers and gradually more intense in the cell layers towards the nymphal chamber)
Starch	Chlorophyllous parenchyma	Centrifugal gradient (weakly in the innermost cell layers and gradually more intense in the cell layers towards the external surface of the gall)
Lipids	Chlorophyllous parenchyma, vascular tissues, cuticle and in the oil glands	Cuticle of the outer epidermis and oil glands
Proteins	Chlorophyllous parenchyma and vascular tissues	Bidirectional gradient (weakly or undetectable in the median cortical cells and gradually more intense in the cell layers towards the internal and external surfaces of the gall)
Enzyme activity	Acid phosphatase; sparsely in the cells of the spongy parenchyma	Glucose-6-phosphatase and invertases; in the cells of vascular tissues and perivascular parenchyma
Total phenolics	Concentrated in the palisade parenchyma; sparsely in the spongy parenchyma	Bidirectional gradient
Terpenoids	Palisade parenchyma and in the oil glands	Bidirectional gradient
Proanthocyanidins	Adaxial surface of the epidermis and in the palisade parenchyma	Bidirectional gradient
Lignins	Mature xylem and fibres	Centrifugal gradient (in the mature xylem, weakly in the inner/median cortical cells and gradually more intense in the outer cortical cells)
ROS	Concentrated in the palisade parenchyma and in the phloem and xylem parenchyma	Bidirectional gradient

#### Enzyme activity

Leaves have detectable activity of acid phosphatase exclusively in the cells of the spongy parenchyma. Galls have detectable activity of glucose-6-phosphatase and invertases in the vascular bundles and perivascular parenchyma of the median and inner cortices (Figs 6E and F and 8; Table 2).

#### Secondary metabolites

**Figure 7.**
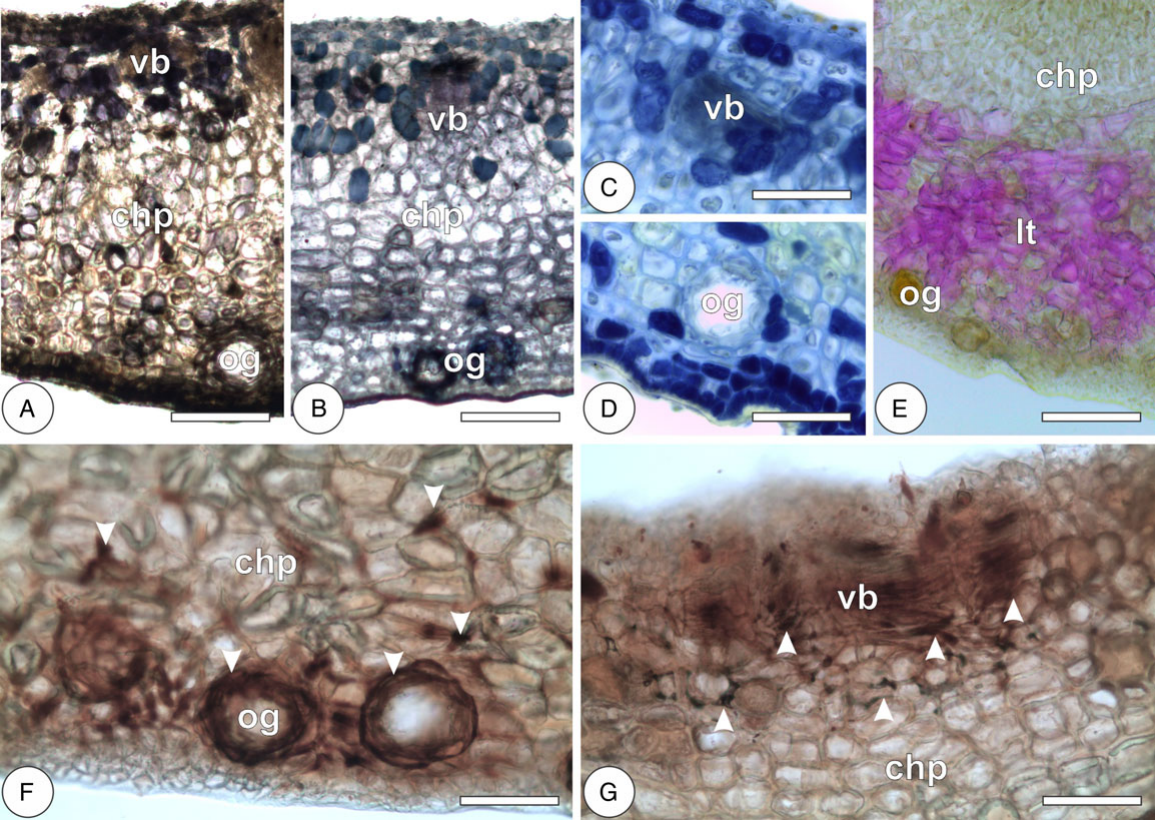
Histochemical detection of secondary metabolites and ROS in the galls of *Nothotrioza cattleiani*. (A–D) Reaction of ferric chloride solution, Nadi and DMACA to total phenolics (A), terpenoids (B) and proanthocyanidins (C and D), respectively. Most intense reactions are observed in the median and inner cortical cells near the vascular bundles (vb), in the outermost cortical cells and in the epidermal cells near the oil glands (og). (E) Reaction of Wiesner's solution to lignins mainly in the outer cortical cells of the chlorophyllous parenchyma (chp), near the oil glands (og). (F and G) Reaction of DAB to ROS mainly in the median and inner cortical cells, vascular bundles (vb) and outermost cortical cells and oil glands (og). Bars: (A, B, and E) 150 µm; (C, D, F and G) 75 µm.

**Figure 8.**
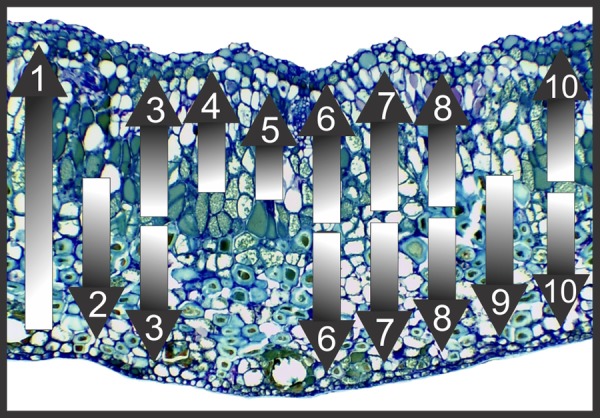
General patterns of histochemical gradients in the cortex of *Nothotrioza cattleiani* galls on *Psidium cattleianum*. Shading from white to dark means increasing intensity of reaction. (1) Centripetal gradient of reducing sugars from the outermost cortical cells towards the innermost ones. (2, 9) Centrifugal gradients of starch and lignins, respectively, from the median cortical cells towards the outer cells. (3, 6, 7, 8, 10) Bidirectional gradients of proteins, phenolics, proanthocyanidins and ROS, respectively, from the median cortical cells towards both the inner and outer cell layers. (4, 5) Centripetal gradients of glucose-6-phosphatase and invertases activities, from the median cortical cells towards the inner cell layers, especially in the perivascular parenchyma.

Phenolic substances and terpenoids are, respectively, evidenced by ferric chloride and Nadi solutions intensely in the palisade parenchyma of the leaves and more scarcely in the epidermis, hypodermis, phloem and spongy parenchyma. Terpenoids are also stained in the epithelium of the oil glands. Proanthocyanidins are detected by DMACA more intensely in the adaxial epidermis and palisade parenchyma and more weakly in the spongy parenchyma. Lignins react with Wiesner's reagent in the cell walls of xylem and periciclic fibres. Galls exhibit bidirectional gradients of phenolic substances, terpenoids and proanthocyanidins from the median cortex towards the inner and outer cortices (Figs 7A–D and 8). Lignins are detected in the secondary walls of the outer cortical cells and in the xylem, forming a centrifugal gradient (Figs 7E and 8; Table 2).

#### Reactive oxygen species

Reactive oxygen species are detected intensely by DAB solution in the chlorophyllous parenchyma of leaves, and also in the phloem and vascular parenchyma. In the galls, ROS are detected weakly in a bidirectional gradient from the median cortex towards the inner and outer cortices. The most intense reactions are observed in the vascular bundles and perivascular parenchyma, and in the epithelium of the oil glands (Figs 7F and G and 8; Table 2).

## Discussion

### Structural–functional implications of cell fates

Cell redifferentiation (*sensu* Lev-Yadun 2003) is a crucial process by which gall-inducing insects coordinate cell reprogramming of plant organs towards gall morphogenesis. In the galls of *N. cattleiani*, this process takes place in the young leaves during the induction phase (*sensu* Carneiro *et al*. 2013), similar to *N. myrtoidis* galls (Carneiro and Isaias 2014; Carneiro *et al*. 2014*a*, *b*). The induction of galls may occur on young and/or mature plant organs, as observed for the leaf galls induced by *Calophya duvauae* on *Schinus polygamus* (Dias *et al*. 2013). The selection of different induction sites affects the patterning of cell fates in gall structure, possibly influencing its adaptive value to the gall inducers (Oliveira and Isaias 2009). Nevertheless, the selective pressures that affect gall morphology seem to be conserved across different gall systems, as convergent types of tissue organization and cell fates, i.e. vascular bundles interspersed with homogenous parenchyma cells, surrounded by sclerenchyma cells, are widely reported in the literature (Meyer 1987; Rohfritsch 1992; Stone and Schönrogge 2003; Oliveira and Isaias 2009; Formiga *et al*. 2011; Carneiro and Isaias 2014; Carneiro *et al*. 2014*a*).

Despite structural similarities, most cell lineages in *N. cattleiani* galls on *P. cattleianum* have developmental dynamics different from those of *N. myrtoidis* galls on *P. myrtoides*. Epidermal cells are the exception to this tendency, as they have conserved cytological traits in both systems, namely vacuolated cells that are well organized in the outer surface of the galls but intermittent in the inner surface (Carneiro and Isaias 2014). Chlorophyllous parenchyma cells in *Nothotrioza* spp. galls have different time-based development. *Nothotrioza myrtoidis* galls have parenchyma cells that develop by standby-differentiation (*sensu* Carneiro and Isaias 2014), in which cell structure remains nearly unaltered for a long period of time, and undergoes major changes from the stage of maturation to senescence. Conversely, the cytology of *N. cattleiani* galls reveals that parenchyma cells gradually and constantly change from induction phase towards senescence. In galls of both *Nothotrioza* spp., the cell walls thicken and lignify in the outermost layers of the cortex, while the photosynthetic and respiratory apparatus of the cells are impaired. Gall structures are assumed to play defensive roles against natural enemies of the galling insects (*sensu* Stone and Schönrogge 2003). The changes of cell fate in the chlorophyllous parenchyma, which have thickened and lignified walls in the galls on *Psidium* spp., suggest the investment in a structure-based defence strategy that should increase the adaptive values of the galls to the *Nothotrioza* spp.

Contrary to the new cell fates of chlorophyllous parenchyma cells in *N. cattleiani* galls, vascular and perivascular parenchyma undergo less structural alteration. The fates of these cells are not altered from non-galled leaves to galls, and they maintain a well-developed metabolic apparatus, namely large nuclei, and dense cytoplasm with abundant polysomes and mitochondria, throughout gall development in both *N. myrtoidis* (Carneiro and Isaias 2014) and *N. cattleiani* galls. The maintenance of structural–functional characteristics of vascular tissues indicates that their primordial role in plants, i.e. conducting water and solutes (Buvat 1989), is maintained in galls. Such a strategy is adaptive (*sensu* Stone and Schönrogge 2003) for the gall inducers as far as their nutrition is concerned, since *Nothotrioza* spp. are sap feeders (Burckhardt 2005; Carneiro *et al*. 2013), and thus dependent on the intake of water and solutes by vascular tissues. Altogether, the cellular characteristics of epidermis, chlorophyllous parenchyma and vascular and perivascular parenchyma corroborate the adaptive nature of the gall structure (*sensu* Stone and Schönrogge 2003), as they directly relate to the maintenance of an adequate microenvironment, protection against natural enemies and enhancement of nutritional options for the gall inducers, as classically hypothesized by Price *et al*. (1987) for galls in general.

In relation to the non-galled leaves, cell fates are completely altered in galls, except for the outer epidermis, whose cell cycles are intensified, but the fates are maintained. In relation to *N. myrtoidis* galls, cell fates are roughly the same, but the standby-redifferentiation observed in the chlorophyllous parenchyma cells during the stage of growth and development (Carneiro and Isaias 2014) does not occur in *N. cattleiani* galls.

### Roles of cell metabolism in gall biology

The functional aspects of plant tissues in the context of gall structure have been related to the accumulation of defensive and nutritive metabolites (Hartley 1998) and to the establishment of histochemical and cytological gradients in Cynipid (Bronner 1992), Cecidomyiidae (Bronner 1992; Oliveira *et al*. 2010, 2011*a*) and Pseudophacopteronidae (Oliveira and Isaias 2010*b*) galls. In the galls of *N. myrtoidis*, such gradients are not observed in gall cortices due to their low structural complexity and metabolism (Carneiro and Isaias 2014). The galls of *N. cattleiani* also lack cytological gradients, as the parenchyma cells are metabolically impaired, with low protein synthesis, scarce and underdeveloped mitochondria and chloroplasts. Nevertheless, the vascular and perivascular parenchyma cells appear increasingly metabolic during gall morphogenesis, i.e. large nuclei, abundant RER, mitochondria, ribosomes and polysomes. Such cell types have also been observed in the vascular and perivascular tissues in galls of *N. myrtoidis* (Carneiro and Isaias 2014) and share great similarities with the nutritive cells of cynipid (Bronner 1992) and cecidomyiid galls (Bronner 1992; Oliveira *et al*. 2010, 2011*b*).

Nutritive tissues have the most specialized cell types within gall structure, and they are constantly affected by the gall inducers during their feeding (Bronner 1992). Both the impact of the feeding activity of the galling insects and the high metabolism intrinsic to these cell types often lead to the accumulation of ROS. In fact, ROS were histochemically localized in the nutritive tissues of galls induced by Cecidomyiidae on *Aspidosperma spruceanum* (Oliveira *et al*. 2010) and on *Copaifera langsdorffii* (Oliveira *et al*. 2011*a*). Furthermore, ROS have been localized in the nutritive-like ground parenchyma around the nymphal chamber in the galls of Pseudophacopteronidae on *A. australe* (Oliveira and Isaias 2010*b*) and in the vascular bundles of *N. myrtoidis* (Carneiro *et al*. 2014*a*) and *E. ostreoides* galls (Isaias *et al*. 2011). Vascular and perivascular parenchyma cells are metabolically active in the galls on *P. myrtoides*, with well-developed ROS-scavenging apparatus, i.e. lomasomes, lamellar and multivesicular bodies (Carneiro and Isaias 2014). Such structures were previously described in the nutritive cells of galls induced by Thysanoptera (Raman and Ananthakrishnan 1983), in the fast-dividing nutritive cells of lepidoptera galls on *Marcetia taxifolia* (Ferreira *et al*. 2015), and also herein for the galls on *P. cattleianum*. Lomasomes, lamellar and multivesicular bodies act together with the endoplasmic reticulum to recycle membrane systems (Staehelin 1997) to ensure the functionality of cells subjected to high oxidative stress.

In addition to the ROS-scavenging apparatus, high protein synthesis seems to be characteristic of nutrition-related tissues in galls, i.e. true nutritive tissues in galls of cynipids (Bronner 1992), cecidomyiids (Oliveira *et al*. 2010, 2011*a*; Ferreira and Isaias 2014) and lepidoptera (Vecchi *et al*. 2013), and of nutritive-like parenchyma cells around the nymphal chamber of Psylloidea galls (Oliveira and Isaias 2010*b*; Isaias *et al*. 2011). In fact, the histochemical detection of proteins in the galls of *N. cattleiani* corroborates such premise and reveals another similarity among vascular and perivascular parenchyma cells in the galls of *P. myrtoides* (Carneiro *et al*. 2014*b*) and *P. cattleianum* and those of true nutritive tissues. The well-developed ROS-scavenging apparatus and high protein synthesis seem to be a widespread trait of nutrition-related cells across different galls. More than simply feeding on the vascular bundle cells, *N. cattleiani* induces the redifferentiation of true nutritive cells in and around the vascular system, which is herein reported for the first time in insect galls.

Other cell types, such as those of the chlorophyllous parenchyma, may also be subjected to high oxidative stress due to photosynthesis and respiration, which are ROS-generating processes (Møller *et al*. 2007). Chloroplasts subjected to high oxidative stress may develop plastoglobules, which help to minimize oxidative damage to the photosynthetic apparatus (Asada 2006; Møller *et al*. 2007). In fact, the chlorophyllous parenchyma cells of non-galled leaves of *P. myrtoides* (Carneiro and Isaias 2014), and of *P. cattelainum* accumulate ROS, and have conspicuous and numerous plastoglobules. Also, they have low-stack grana with electron-lucent thylakoids, which is characteristic of sun-type chloroplasts previously described by Lichtenthaler *et al*. (1981), which photosynthesize at high levels. Galls that accumulate high amounts of ROS, such as the ones induced by the sucking insect *Pseudophacopteron* sp. on *A. australe* (Oliveira *et al*. 2011*b*), have plastoglobules in the chloroplasts. In this system, the stress imposed by the galling herbivore stimulates the differentiation of plastoglobules, which are not observed in non-galled leaves, and both galls and leaves photosynthesize at the same levels (Oliveira *et al*. 2011*b*). In the galls of *N. cattleiani*, the differentiation of plastoglobules is blocked, similar to the galls of *N. myrtoidis* (Carneiro and Isaias 2014), which are photosynthesis deficient (Carneiro *et al*. 2014*b*). In both *Nothotrioza* spp. galls, the chloroplasts have less lamellation throughout gall development and high-stack grana with electron-dense thylakoids, as described for shade-type leaves (Lichtenthaler *et al*. 1981), which photosynthesize at low levels. The cytological features of chloroplasts in the chlorophyllous parenchyma of *N. cattleiani* galls indicate low cellular metabolism and corroborate the hypothesis that these galls may be photosynthesis-deficient, such as the ones of the co-generic system, *N. myrtoidis* (Carneiro and Isaias 2014; Carneiro *et al*. 2014*b*).

Despite the low metabolism of chlorophyllous parenchyma cells in the galls of *N. cattleiani*, secondary and primary metabolites are histochemically localized in the cortex of mature galls, forming gradients somewhat similar to those reported for *N. myrtoidis* galls (Carneiro *et al*. 2014*b*). In both *Nothotrioza* spp. galls, the scarcity of lipid droplets and essential oils is attributed to the characteristics of the host plants, *P. myrtoides* and *P. cattleianum*. These compounds are common in Myrtaceae (Ramos *et al*. 2010) and represent a metabolic trait that is not changed due to gall formation, as observed for galls of *Aceria lantanae* on *Lantana camara* (Verbenaceae) (Moura *et al*. 2008). The centripetal gradients of reducing sugars, on the other hand, evidence the manipulation of plant cell metabolism in galls, which conspicuously accumulate sugars. Sugars are not synthesized at gall sites, but they are drained from non-galled organs (Burstein *et al*. 1994; Raman *et al*. 2006; Castro *et al*. 2012*b*, 2013). Herein, this premise is confirmed for the galls of *N. cattleiani*, which have detectable activity of glucose-6-phosphatase and invertases in the vascular bundles and surrounding tissues, next to the larval chamber. These sites of enzyme activity are similar to those described in the galls on *A. australe* (Oliveira and Isaias 2010*b*), *A. spruceanum* (Oliveira *et al*. 2010) and *Lonchocarpus muehlbergianus* (Isaias *et al*. 2011). Glucose-6-phosphatase is involved in the formation of sucrose after starch breakdown (Baroja-Fernández *et al*. 2003) and invertases catalyse the irreversible conversion of sucrose into glucose and fructose (Koch 2004). The centrifugal histochemical gradient of starch in the galls of *P. cattleianum* indicates that starch is converted into soluble sugars in the cells surrounding the larval chamber, where enzyme activity is detected. A similar gradient of starch and related enzyme activity is described for the galls on *L. muehlbergianus* (Isaias *et al*. 2011), which reinforces the enzyme-mediated mobilization of starch, as a pattern for inner cortical cells of galls. The activities of enzymes related to carbohydrate metabolism determine the establishment of physiological sinks in plants (Koch 1996; Koch and Zeng 2002) and are involved in tissue development and cell expansion (Rehill and Schultz 2003), crucial for gall development. In fact, the galls on *Psidium* spp. act as physiological sinks, but diverge in terms of the detectable carbohydrate-related enzymes. The activity of acid phosphatase is exclusive of *P. myrtoidis* galls (Carneiro *et al*. 2014*b*), while the activity of glucose-6-phosphatase and invertases is detected only in *P. cattleianum* galls.

Besides accumulating carbohydrates, the galls on *P. cattleianum* also have gradients of total phenolics and proanthocyanidins, similar to the galls on *P. myrtoides* (Carneiro *et al*. 2014*b*). The localization of such compounds in gall tissues is considered a defensive chemical strategy of gall inducers against natural enemies (Bronner 1992) for their unpalatability. In both galls, phenolic compounds are believed to control cell expansion and division by the modulation of indol-acetic acid (IAA) levels (Hori 1992). In fact, recent studies localized phenolics and IAA at the same sites in *Piptadenia gonoacantha* galls (Bedetti *et al*. 2014), thus confirming the developmental role of phenolics in galls. Also, phenolics mediate the morphogenesis of vascular tissues (Aloni 2001) and should be involved in the neoformation of vascular bundles near the nymphal chamber. Neoformed vascular bundles contribute to the establishment of the gall as a sink of photoassimilates and to the nutrition of the gall inducer, which contradicts the classical role of phenolics as plant-defensive compounds in galls, as widely attributed in literature (Hartley 1998; Nyman and Julkunen-Tiitto 2000; Formiga *et al*. 2009). Another group of phenolic substances, the proanthocyanidins, accumulates both in the outermost and innermost cell layers of the *Psidium* spp. galls, with decreasing gradients towards the median cortex. Such substances act as antioxidants (Simmonds 2003; Bouaziz *et al*. 2005), and their distribution along the gradient of ROS concentration possibly prevents oxidative stress in gall tissues, keeping cell alterations to a minimum.

## Conclusions

The similar phenotypes of the globoid galls of the double co-generic systems, *N. cattleiani*–*P. cattleianum* and *N. myrtoidis*–*P. myrtoides*, are not extended to the cytological and histochemical levels. Nevertheless, the lack of cytological gradients and the formation of somewhat conserved histochemical profiles are common to both galls, with the centripetal accumulation of sugars and centrifugal detection of lignins, as conserved traits possibly linked to the taxonomical proximity of the involved species.

The bidirectional gradients of metabolite accumulation described for *N. cattleiani* galls, together with the redifferentiation of true nutritive cells associated with vascular bundles, are unique features. Also, cytological development in *N. cattleiani* galls is gradual from induction towards senescence, and different from the standby-redifferentiation of *N. myrtoidis* galls, which reinforce the species-specific subcellular aspects of these distinctive extended phenotypes.

## Sources of Funding

Our work was funded by the Conselho Nacional de Desenvolvimento Científico e Tecnológico (Brazil) (grant number 307007/2012-2), the Empresa Brasileira de Pesquisa Agropecuária (Project: ‘Manejo e biodiversidade de Psylloidea associados ao sistema integração lavoura—pecuária—floresta e à citricultura no Brasil’, number 02.12.01.028.00.00), Fundação de Amparo a Pesquisa do Estado de Minas Gerais (Brazil) and Coordenação de Aperfeiçoamento de Pessoal de Nível Superior (Brazil).

## Contributions by the Authors

Both authors substantially contributed to the preparation of manuscript and the research presented; they have seen and agreed to submit the manuscript.

## Conflict of Interest Statement

None declared.
